# Involvement of glutamine synthetase 2 (*GS2*) amplification and overexpression in *Amaranthus palmeri* resistance to glufosinate

**DOI:** 10.1007/s00425-022-03968-2

**Published:** 2022-08-12

**Authors:** Matheus M. Noguera, Aimone Porri, Isabel S. Werle, James Heiser, Frank Brändle, Jens Lerchl, Brent Murphy, Michael Betz, Fanny Gatzmann, Martin Penkert, Clara Tuerk, Lucie Meyer, Nilda Roma-Burgos

**Affiliations:** 1 Department of Crop, Soil, and Environmental Sciences, University of Arkansas, 1371 W Altheimer Dr, Fayetteville, AR 72704 USA; 2 BASF SE, Agricultural Research Station, Limburgerhof, Germany; 3 Fisher Delta Research Center, University of Missouri, Portageville, MO USA; 4 IDENTXX GmbH, Stuttgart, Germany; 5 Present Address: Department of Crop Sciences, University of Illinois, Champaign, USA

**Keywords:** Gene amplification, Gene expression, Glufosinate resistance, Glutamine synthetase, Palmer amaranth

## Abstract

**Main conclusion:**

Amplification and overexpression of the target site glutamine synthetase, specifically the plastid-located isoform, confers resistance to glufosinate in *Amaranthus palmeri*. This mechanism is novel among glufosinate-resistant weeds.

**Abstract:**

*Amaranthus palmeri* has recently evolved resistance to glufosinate herbicide. Several *A. palmeri* populations from Missouri and Mississippi, U.S.A. had survivors when sprayed with glufosinate-ammonium (GFA, 657 g ha^−1^). One population, MO#2 (fourfold resistant) and its progeny (sixfold resistant), were used to study the resistance mechanism, focusing on the herbicide target glutamine synthetase (GS). We identified four *GS* genes in *A. palmeri*; three were transcribed: one coding for the plastidic protein (*GS2*) and two coding for cytoplasmic isoforms (*GS1.1* and *GS1.2*). These isoforms did not contain mutations associated with resistance. The 17 glufosinate survivors studied showed up to 21-fold increase in *GS2* copies. *GS2* was expressed up to 190-fold among glufosinate survivors. *GS1.1* was overexpressed > twofold in only 3 of 17, and *GS1.2* in 2 of 17 survivors. GS inhibition by GFA causes ammonia accumulation in susceptible plants. Ammonia level was analyzed in 12 F1 plants. *GS2* expression was negatively correlated with ammonia level (*r* =  – 0.712); therefore, plants with higher *GS2* expression are less sensitive to GFA. The operating efficiency of photosystem II (ϕPSII) of *Nicotiana benthamiana* overexpressing *GS2* was four times less inhibited by GFA compared to control plants. Therefore, increased copy and overexpression of *GS2* confer resistance to GFA in *A. palmeri* (or other plants). We present novel understanding of the role of GS2 in resistance evolution to glufosinate.

**Supplementary Information:**

The online version contains supplementary material available at 10.1007/s00425-022-03968-2.

## Introduction

*Amaranthus palmeri* S. Watson (Palmer amaranth) is a summer annual forb native of the Sonoran Desert (Ehleringer 1983), which encompasses large regions of the southwestern United States and northwestern Mexico. Due to the globalization of agricultural markets and new habitat creation through agriculture expansion, this species has been introduced to several other countries and now can be found in all continents (Roberts and Florentine 2021). Its biology, physiological characteristics and impressive adaptation potential has made this species a major threat to food security and the preservation of native ecosystems and wildlife (Ward et al. 2013; Roberts and Florentine 2021). Climate change is likely to favor its establishment and expansion into key row-crop areas worldwide that are currently free of *A. palmeri*, or have incipient infestations, and enhance its competitive ability against crops (Kistner and Hatfield 2018; Briscoe Runquist et al. 2019).

The adaptability of *A. palmeri* is demonstrated by its propensity to evolve resistance to herbicides. With resistance to nine sites of action (SoA) reported, *A. palmeri* is only behind *Lolium rigidum* globally, with the latter having resistance to 12 SoA (Heap 2022). Resistance traits can accumulate in a plant. For instance, resistance to six SoA was reported in a genotype from Kansas, U.S.A. (Shyam et al. 2021). This characteristic reduces the already limited herbicide options for *A. palmeri* and hinders its management.

The latest addition to the list of herbicides to which *A. palmeri* has evolved resistance is glufosinate (Heap 2022). This active ingredient is a glutamic acid analog, known by its fast, non-selective activity and reduced risk from the toxicological and environmental standpoints (Zhou et al. 2020; Duke et al. 2022). Glufosinate is mostly formulated as ammonium-salt (hence, glufosinate-ammonium or GFA), and only the L-isomer has herbicidal activity (Hoerlein 1994). The racemic mixture is commercially preferable due to lower production cost.

Glutamine synthetase (GS, EC 6.3.1.2), the target site of GFA, is an essential enzyme that catalyzes the ATP-dependent incorporation of ammonia to glutamate, yielding glutamine (Miflin and Habash 2002). This reaction is the first step of N assimilation in plants, which also involves glutamate synthase (GOGAT, EC 1.4.1.13), to drive the GS/GOGAT cycle (Masclaux-Daubresse et al. 2010). The GS/GOGAT cycle also produces glutamate to serve as an N donor for the synthesis of glycine from glyoxylate, derived from photorespiration (Dellero et al. 2016). GS inhibition causes an accumulation of ammonia, glycolate and glyoxylate, inhibiting photosynthesis and leading to a state of extreme oxidative stress in the presence of light, which causes cell and plant death (Oliver 1980; Sauer et al. 1987; Campbell and Ogren 1990; Coetzer and Al-Khatib 2001; Takano et al. 2020).

Resistance to GFA has evolved slower than to many herbicides with different sites of action (SoAs). In 2009, *Eleusine indica* was the first species to be reported as GFA-resistant (Jalaludin et al. 2010). After that, resistance to GFA was documented in two species from the *Lolium* genus, and *A. palmeri* is the first dicot weed to evolve resistance to GFA (Heap 2022). The only resistance mechanisms reported so far were the increased GFA metabolism in a *Lolium perenne* var. *multiflorum* (Brunharo et al. 2019) and the S59G mutation in the *GS1-1* gene from *E. indica* (Zhang et al. 2022). Resistance mechanism has yet to be determined in the remaining cases. Widespread weed resistance to the non-selective herbicide glyphosate has increased the use of this alternative non-selective herbicide glufosinate, increasing the selection pressure on weed species. The recent evolution of resistance to GFA in *A. palmeri* is a testament to that. The objectives of this research were to: (1) assess the level of GFA resistance in a selected population; (2) determine if resistance is heritable; and (3) identify the mechanism(s) conferring resistance.

## Materials and methods

### Plant materials, growth conditions, and application parameters

The putative glufosinate-resistant Palmer amaranth population was collected from a soybean farm in Butler County, Missouri, at the end of 2020 growing season. The sensitive standard (SS) accession was collected in Crawford County, Arkansas, from a field with a history of minimal herbicide use. Sampling and collection were done according to standard protocols (Burgos 2015). To generate the F1 population, ten plants from the MO#2 population that survived an application of 657 g ha^−1^ GFA were transplanted to 8L pots and grown together in a greenhouse until maturity. Female inflorescences were harvested, threshed and seeds were cleaned and stored in glass vials.

Plants were grown in a greenhouse maintained at 32/28 °C day/night temperature and a photoperiod of 14 h achieved with supplemental light. Irrigation was done via capillarity as needed and plants were fertilized once a week using a diluted water-soluble, all-purpose plant food (Miracle-Gro, 15-30-15 NPK).

Herbicide applications were done using a benchtop sprayer, equipped with two Teejet Flat Fan 110 0067 nozzles, calibrated to deliver 187 L ha^−1^ of spray mix at 3.6 km h^−1^ and 275 kPa. Nozzle spacing was 50 cm and boom height was set to 45 cm above the plant canopy.

### Response of MO#2 and its progeny to glufosinate

Seeds were sown in 50-cell trays filled with a commercial potting mix (Sun Gro Horticulture, Agawam, MA, USA) and seedlings were thinned to 1 plant per cell a week after emergence. When plants were 5 to 8-cm tall, 7 rates of glufosinate (Liberty 280 SL, BASF SE, Ludwigshafen, Germany) were sprayed. Putative resistant populations (MO #2 and its progeny) were sprayed with 82, 164, 328, 657, 1314, 2628 and 5256 g ai ha^−1^ (corresponding to 0.125 × , 0.25 × , 0.5 × , 1 × , 2 × , 4 × and 8 × of the labeled rate). The SS was sprayed with 5, 10, 20, 41, 82, 164 and 328 g ai ha^−1^ (covering 0.0078 × to 0.5 × the labeled rate). The 1 × GFA rate is the herbicide label rate of 657 g ai ha^−1^. The adjuvant ammonium-sulphate was added to all treatments at 10 g L^−1^ of spray mix. A nontreated check was included for all populations, two replications were used per treatment (1 rep = 25 plants), and the test was conducted twice. To avoid time-of-day effects on herbicide activity, both runs were sprayed from 1 to 2 PM. Applications of GFA during full sunlight tend to provide better weed control (Martinson et al. 2005). Fifteen days after treatment (DAT), live plants were counted and the data were converted to survival percentage. Survival data was fitted to a non-linear regression as described in the “Statistical analysis” section.

### GS isoforms identification in *A. palmeri* genome

Gene annotation files of the *A. palmeri* genome (Montgomery et al. 2020) were parsed and four sequences were retrieved: g13234, g1417, g17049 and g17050. Upon comparison of their peptide sequences with 34 publicly available sequences representative of different plant families (from Phytozyme and Genbank databases, Supplementary Fig. S1 and File F1), it was determined that g13234 and g1417 (hereafter called *GS1.1* and *GS1.2*, respectively) encoded the cytosolic isoforms, whereas g17049 and g17050 (hereafter called *GS2.1* and *GS2*, respectively) encoded the plastidic proteins. A phylogenetic tree was built using the 34 sequences retrieved from online databases in addition to the sequences from *A. palmeri*. The tree was done using Geneious Prime software (Biomatters) and the neighbor-joining method, with no outgroups.

Because GS2 in plants is usually coded by a single nuclear gene, the two plastidic isoforms found in *A. palmeri* were further investigated by extracting a 40-kb surrounding genomic region and constructing a synteny dot plot using kmers (*k* = 10), where the region was compared to itself to identify genomic signatures of duplication and conservation.

### Homology modeling

To identify the residues involved in GFA binding into GS1.1, the protein crystal structure of *Zea mays* GS1 (PDB 2D3A) was used as a template to build a homology model for *A. palmeri*. L-glufosinate was docked into the GS1.1 binding site. To guide the docking, we used the GFA binding mode from the protein crystal structure of *Salmonella* (1FPY). Molecular modeling was done using Molecular Operating Environment (MOE) 2020.09 software package (Chemical Computing Group ULC 2022).

### Sequencing of GS isozymes from the GFA survivors

#### RNA extraction and cDNA synthesis

Leaf Sects. (0.5 cm^2^) were sampled, transferred into a collection microtube (Qiagen) and snap-frozen in liquid nitrogen. Samples were homogenized with steel beads in a shaker mill (TissueLyser II; Qiagen) and total RNA was extracted in a magnetic particle processor (Thermo Fisher Scientific, Waltham, MA, USA) using the MagMAX™ Plant RNA Isolation Kit (Applied Biosystems) according to the manufacturer's instructions. An aliquot of 200 ng of total RNA was used for cDNA synthesis using the High-Capacity cDNA Reverse Transcription Kit (Applied Biosystems) according to the manufacturer's instructions.

#### Preparation of tailed cDNA for RACE PCR

The cDNA for RACE PCR was prepared using the SMARTer® RACE 5’/3’ Kit (Takara Bio Europe) according to the manufacturer's instructions. In brief, 10 µL of total RNA (200 ng/µL) was incubated with 1 µL of 5'-CDS Primer A for the 5' tailed cDNA or with 1 µL of 3'-CDS Primer A for the 3' tailed cDNA at 72 °C. After 3 min, the temperature was decreased to 42 °C for 2 min. In addition, 1 µL of the SMARTer II A Oligonucleotide was added to the 5'-RACE preparation. The 3'-RACE preparation was used directly. To these solutions were added 4.0 µL of 5X First-Strand Buffer, 0.5 µL of dithiothreitol (DTT, 100 mM), 1.0 µL of dNTPs (20 mM), 0.5 µL of RNase inhibitor (40 U/µL), and 2.0 µL of SMARTScribe Reverse Transcriptase (100 U). Reverse transcription was performed at 42 °C for 90 min. After heat inactivation for 10 min at 70 °C, the tailed cDNA was used for RACE PCR.

#### RACE PCR

For RACE PCR, cDNA was amplified in a 25-µL reaction containing 1 µL (10 pmol) of specific RACE primers, 2 µL of the Universal Primer A Mix, 12.5 µL SNP Pol 2X PCR Master Mix (Genaxxon bioscience GmbH, Ulm, Germany) 4.5 µL PCR-Grade H_2_O and 5 µL of the tailed cDNA. The RACE PCR performed in a thermal cycler (T100, Bio-Rad Laboratories) under the following conditions: 3 min at 94 °C and 42 cycles of 10 s denaturation at 94 °C; 35 s annealing at 68 °C and 3 min elongation. Aliquots were taken and analyzed on 1.5% agarose gels. Bands of the expected size were cut out and cleaned (innuPREP DOUBLEpure Kit, IST Innuscreen GmbH, Berlin, Germany).

The PCR products were verified with specific nested primers under the following conditions: 3 min at 94 °C and 35 cycles of 10 s denaturation at 94 °C; 35 s annealing at 65 °C and 90 s elongation at 72 °C; and a final elongation step at 72 °C for 5 min. Aliquots were taken and analyzed on 1.5% agarose gels. Bands of the expected size were cut out, cleaned, and subsequently cloned using StrataClone PCR Cloning Kit (Agilent). Positive white colonies were randomly picked and verified with colony PCR. For each clone, 10 positive PCR fragments were randomly selected and verified via Sanger sequencing (SeqLab-Microsynth, Göttingen, Germany). Sequences were analyzed using Geneious Prime software v. 9.1.8 (Biomatters).

#### End point PCR for entire coding sequences

Full-length amplification of GS coding sequences was performed in a final volume of 25 µL reaction, composed of 5 µL of cDNA, 1 µL (10 pmol) of F and R primers (Table S1), 12.5 of MyFi™ DNA Polymerase (Bioline GmbH) and 6.5 µL of H_2_O. Amplification was done in a thermal cycler (T100, Bio-Rad Laboratories) under the following conditions: 3 min at 95 °C and 35 cycles of 10 s denaturation at 95 °C; 35 s annealing at primer-specific temperature (Table S1) and 2 min elongation at 72 °C, followed by a final elongation step at 72 °C for 5 min. Aliquots were taken and submitted to gel electrophoresis to confirm the presence of a single amplicon. PCR products were Sanger-sequenced (SeqLab-Microsynth) and results were analyzed using Geneious Prime software v. 9.1.8 (Biomatters).

### GS copy number and expression analysis

Seventeen GFA survivors from the MO #20 population were sampled at 3 weeks after application for GS copy number and expression analysis. A 0.5-cm^2^ leaf tissue was transferred into a collection microtube (Qiagen) and homogenized in a shaker mill (Qiagen) with steel beads. DNA extraction was performed in magnetic particle processors (KingFisher™, Thermo Fisher Scientific) using the Chemagic Plant 400 kit (Perkin Elmer, Waltham, MA, USA) according to the manufacturer's instructions (modified by IDENTXX GmbH, Stuttgart, Germany). RNA extraction and cDNA synthesis were done as described in Sect. 2.5.1.

TaqMan™ assays were designed to allow a multiplex approach for the target and reference genes. GS1 isoforms plus Actin genes were run in a triplex reaction, while GS2 was run in duplex with Actin, and each sample was run in triplicate. Gene expression and copy number were assayed using cDNA and gDNA as templates, respectively.

qPCR assays were performed in a 25 µL reaction composed of 5 µL of cDNA/gDNA, 1 µL (0.2 µM) of primers and 0.25 µL (0.2 µM) of probe, 0.25µL of SNP PolTaq DNA Polymerase and 2.5 µL 10X buffer (Genaxxon bioscience), 0.5 µL dNTP mix (10 mM) and 13 and 14.25 µL H_2_O for the triplex and duplex qPCR, respectively. Reactions were performed in a qPCR thermal cycler (Bio-Rad Laboratories) under the following conditions: 5 min at 95 °C, and 35 cycles of 95 °C for 10 s and 60 °C for 30 s. Real-time fluorescence data were captured during the amplification cycle.

### Ammonia accumulation assay

Ammonia accumulation after GFA application has been used as an indicator of plant susceptibility to this herbicide, in both crops (Pornprom et al. 2003; Domínguez-Mendez et al. 2019) and weeds (Avila-Garcia et al. 2012; Salas-Perez et al. 2018). To verify if GS2 fold-change in expression correlates with ammonia levels, an in vitro assay was done using a modified methodology described by Dayan et al. (2015). In this assay, 12 survivors from the MO#2 F1 population were used and sampling occurred at 5 weeks after application. Briefly, three leaf discs (5 mm diameter) were cut from the youngest fully expanded leaf of each plant and placed in a microplate containing 150 uL of a 20-uM GFA (bathing) solution. Each well contained a single leaf-disc and represented a replication. The plate was sealed with two layers of micropore tape and kept in a growth chamber under continuous light at 28 °C for 24 h. The reaction was stopped by placing the plate at – 80 °C. After two freeze–thaw cycles, a 50-uL aliquot of the bathing solution was transferred to a fresh plate for ammonia quantification as described by Molin and Khan (1995). Absorbance at 630 nm was read using a microplate reader (SpectraMax iD3, Molecular Devices LLC, San Jose, CA, USA) and converted to mM NH_4_^+^ g fresh biomass^−1^ using a standard curve produced with ammonium chloride.

### GS isoforms quantification

To check if the higher number of GS copies and transcripts observed in resistant plants would result in higher protein levels, the three GS isoforms were quantified in the same 12 plants used in the previous study. Leaf samples were collected around 3 months after GFA application. For this reason, the assays for GS copy number and expression, which were done on these same plants 24 h after GFA application, were conducted again on these samples.

#### Protein extraction

Sampling was done by collecting and pooling the youngest fully expanded leaves from different branches into a 50-mL Falcon tube and immediately freezing it in liquid nitrogen. Samples were ground in liquid nitrogen using a mortar and pestle, and 400 mg of leaf powder was mixed with lysis buffer (5% SDS; 50 mM TEAB; pH = 8.5) and incubated at 70 °C for 10 min. After centrifugation at 20,000 g for 10 min, the remaining supernatant was filtered (0.45-µm filter). Total protein was quantified using the Pierce™ 660 nm kit (Thermo Fisher Scientific) and concentration adjusted to 150 µg per sample.

#### Protein digestion and peptide clean-up

Protein digestion and peptide clean-up was done using the S-trap™ micro spin columns kit (ProtiFi LLC, Farmindale, NY, USA) as per manufacturer instructions. In brief, reduction was conducted by adding dithiothreitol (DTT) to a final concentration of 20 mM and incubating at 60 °C for 10 min. Alkylation was performed by addition of IAA to a final concentration of 60 mM and incubation in the dark at room temperature for 30 min. For protein digestion, 22 µL 12% H_3_PO_4_ plus 725 µL S-Trap™ binding buffer were added. The solution was loaded onto a S-Trap™ Micro column and washed four times with the binding buffer. Digestion was carried out for 1 h with 1.5 µg Lys-C and overnight with 3 µg trypsin diluted in 100 µL digestion buffer (50 mM TEAB). Elution of digested peptides was mediated by centrifuging for 1 min. Within two steps 40 µL of 0.2% FA and 40 µL of 0.2% FA in 50% ACN solution were loaded onto the column and centrifuged at same conditions. The flow through was vacuum-dried and dissolved in 100 µL of 1% FA. Desalting of the digested protein samples was performed by SDB Stage Tip purification. SDB Stage Tips were conditioned with 100 µL methanol and 100 µL SDB Stage Tip buffer B (80% ACN, 0.1% FA) and 2 × 100 µL SDB Stage Tip buffer A (0.1% FA). Samples were loaded and washed two times with 200 µL of SDB Stage Tip buffer A and 200 µL of SDB Stage Tip buffer. Elution was performed with 20 µL of elution buffer (5% NH_4_OH in 60% ACN, pH > 9). The eluate was collected and vacuum dried. For mass spectrometry measurement, the dried sample was taken up in 100 µL of 0.1% FA and 2% acetonitrile in water.

#### nanoLC–MS/MS analysis

Three technical replicates per sample were analyzed by a reverse-phase nano-liquid chromatography system (EASY-Spray™ 1200, Thermo Fisher Scientific) connected to an Orbitrap Fusion™ mass spectrometer (Thermo Fisher Scientific). LC separations were performed on a 25 cm × 75 μm, C18 “Aurora” column (IonOpticks, Melbourne, VIC, Australia) packed with 1.7-μm particles at an eluent flow rate of 300 nL min^−1^ using a gradient of 2 to 17% B in 72 min, 17 to 27% B in 28 min and 27–41% B in 20 min. Mobile phase A contained 0.1% FA and 2% acetonitrile in water, and mobile phase B consisted of 0.1% FA in 80% acetonitrile in water. Fourier transformed survey scans were acquired in a range of m z^−1^ 375 to 1500 with a resolution of 240,000 at an automatic gain control target of 100% and a max injection time of 50 ms. In data-dependent mode monoisotopic precursor ions with charge states between 2 and 7 were selected for fragmentation. HCD MS/MS spectra were acquired in the ion trap with a normalized collision energy of 35%, an automatic gain control target of 20% and a dynamic max injection time. Fragmented precursor ions were dynamically excluded from fragmentation for 20 s.

Raw data were search by MaxQuant 2.0. (Tyanova et al. 2016) against an inhouse database for *A. palmeri* containing the different GS variants. Default MaxQuant parameters were used. Trypsin was chosen for digestion allowing up to two missed cleavages. N-terminal acetylation and methionine oxidation were considered as variable modifications and carbamidomethylation of Cys was specified as fixed modification. The false discovery rate was set to 1% for both peptide spectrum level and protein level. Label-free quantification (LFQ) including the match-between runs feature was enabled and LFQ min ratio count was set to 2. At least two unique peptides were considered for quantification. A fold-change in protein levels was calculated by dividing the LFQ intensity of the sample by the average LFQ intensity of three plants from the SS population.

### Nicotiana benthamiana leaf infiltration with A. palmeri GS2

To provide further evidence that *GS2* overexpression can lead to GFA resistance, transient expression of *A. palmeri GS2* in *N. benthamiana* was done using the leaf infiltration technique (Sparkes et al. 2006), and leaf discs were incubated in a GFA solution. The operating efficiency of photosystem II (ϕPSII) was used as an indicator of photosynthetic activity in response to GFA (Murchie and Lawson 2013).

A plasmid containing *A. palmeri GS2* was inserted into an *Agrobacterium* strain and cultured. The culture was centrifuged for 20 min at 3000 g at 22 °C, and the pellet was washed with 50 mL H_2_O. After another centrifugation step, 20 mL of an infiltration medium (10 mM MgCl_2_, 10 mM MES pH 5.2, 10 µM acetosyringone) was used to re-suspend the pellet to OD600 = 1. The solution was incubated for 2 h at room temperature. The abaxial surface of *N. benthamiana* leaves were infiltrated using a needleless 1-mL syringe and incubated for 7 d at 23 °C. Control plants were infiltrated with the empty plasmid. Leaf discs (8 mm diameter) were sampled from control and transformed plants, and individually placed in the wells of a microtiter plate containing GFA solution (prepared with technical grade GFA and Milli-Q water). Nine rates were used ranging from 1 µM to 10 mM, and each rate was placed in three wells. Control treatments had water only. After 48 h of incubation in the herbicide solution, ϕPSII was measured using a DUAL-PAM-100 (Heinz Walz GmbH, Effeltrich, Germany). Data was converted to percentage inhibition relative to control plants and fitted with a non-linear regression as shown in Sect. 2.10.

### Statistical analysis

Dead or alive counts from the dose–response experiment were transformed to survival percentage. A three-parameter log-logistic model was then fitted to the data (Ritz et al. 2016) using the package “drc” in R 4.0.3 (R Core Team 2019), as shown in Eq. 1. To assess fitness of the model, a lack-of-fit test was done using the modelFit function from the drc package.

1 $$Y = \frac{d}{{1 + \exp (b(\log x - \log ED_{50} ))}}$$

In Eq. 1, Y is the percent survival, *d* is the upper asymptote, *x* is the GFA rate, and *b* is the slope around ED_50_, which is the value of *x* giving a 50% response of Y. Differences in ED_50_ among populations were evaluated using the compParm function, and resistance index was calculated by dividing ED_50_ R/ED_50_ SS. Confidence intervals of the ED_50_ were estimated using the ED function. Similarly, the operating efficiency of photosystem II (ϕPSII) of *N. benthamiana* samples was converted to percent inhibition relative to control plants and fitted with a 4-parameter Weibull II model (Eq. 2).

2 $$Y = c + \left( {d - c} \right)\left[ {1 - \exp \left( { - \left( {{\raise0.7ex\hbox{$x$} \!\mathord{\left/ {\vphantom {x e}}\right.\kern-\nulldelimiterspace} \!\lower0.7ex\hbox{$e$}}} \right)^{b} } \right)} \right]$$

In Eq. 2, *Y* is the percent inhibition, *x* is the herbicide concentration, *c* and *d* are the lower and upper asymptotes, respectively, and *b* is the slope around *e*, which is the inflection point of the dose–response curve. The I_50_ (dose of GFA required to cause a 50% reduction in Y) was estimated for the samples overexpressing GS2 and the empty vector, and compared using the compParm function in drc.

Gene expression and copy number analysis was dose using the 2^–∆∆Ct^ method (Schmittgen and Livak 2008) using the software CFX Maestro 2.2 (Bio-Rad Laboratories). Dose–response graphs were done using the drc package in R, and all other graphs were generated using SigmaPlot 14.5 (Systat Software, Inc.).

## Results

### Resistance level of a GFA-resistant *A. palmeri* from Missouri

**Fig. 1:**
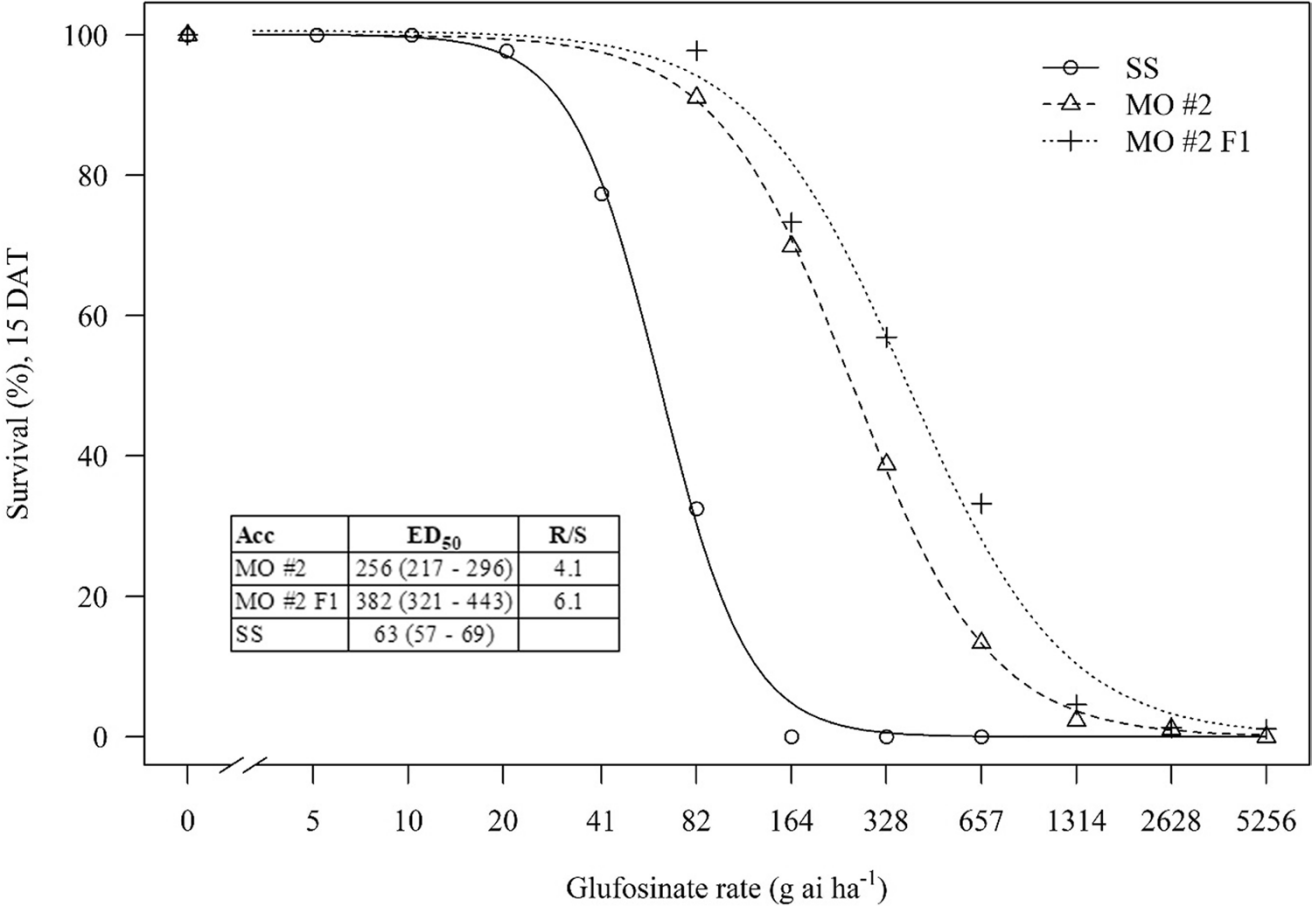
Response of MO #2, MO #2 F1 and SS to increasing rates of GFA. Labeled GFA rate is 657 g ha^−1^. Percent survival data was fitted with a three-parameter log-logistic model and ED_50_ (GFA rate that controls 50% of plants) was estimated for each population. Confidence intervals of these parameters are shown between brackets. Data points are means of two runs with four replications per treatment (total *n* = 8)

None of the resistant populations were controlled 100% at the labeled herbicide rate (1x = 657 g ai ha^−1^), whereas the SS was completely controlled at ¼x (Fig. 1). Early herbicide symptoms (leaves with water-soaked appearance) were observed as soon as 1 h after treatment (HAT), with severe necrosis developing from 24 HAT onwards. The estimated ED_50_ for MO#2 and MO#2 F1 were 256 and 381 g ai ha^−1^, respectively, which were equivalent to 4.1- and 6.1-fold resistance index, respectively, compared to SS.

### Identification of GS isoforms in *A. palmeri* and herbicide-binding residues

**Fig. 2:**
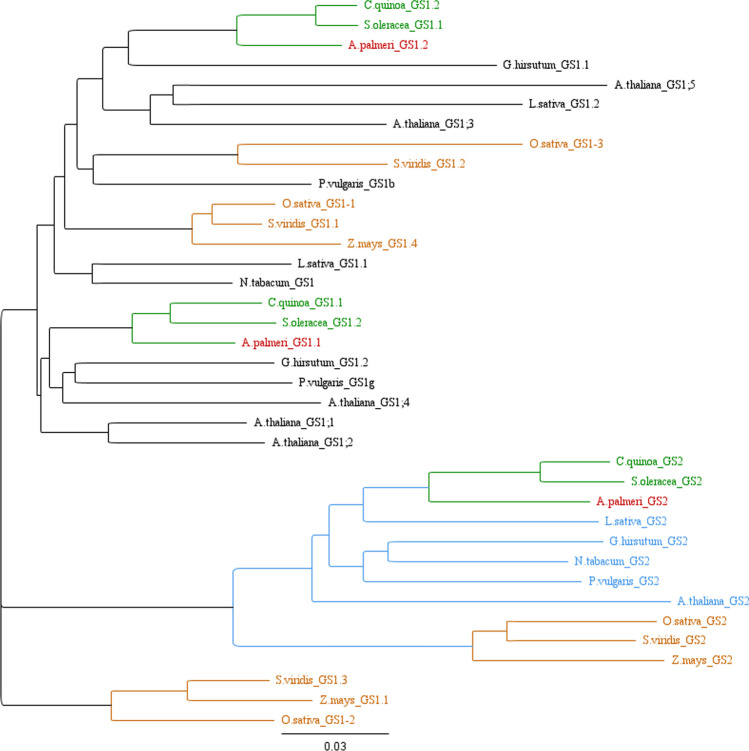
Phylogenetic tree composed of 37 *GS* isoforms from 12 plant species. Multiple alignment and tree construction were performed using Geneious Prime. *A. palmeri* isoforms are highlighted in red and clustered with other species from the Amaranthaceae family (green clades). GS isoforms in grasses were closely related (orange clades). All GS2 sequences clustered in a well-defined clade (blue). Sequences were obtained from Phytozeme and Genbank databases and entries are shown in the Supplementary file F1

The *A. palmeri* genome carries two cytosolic isoforms (*GS1.1* and *GS1.2*) and two chloroplastic isoforms (*GS2.1* and *GS2*). Phylogenetic analysis of GS isoforms from 11 species showed a close relation between *A. palmeri* isoforms and its homologs in other species from the *Amaranthaceae* family (Fig. 2). The *GS2.1* gene was located adjacent to *GS2* in the *A. palmeri* genome. At the protein and mRNA level, these two genes show a large degree of conservation as shown in the BLAST output, where 00,779 (g17050) was used as the query. However, once the genomic level was assayed, the association fell apart and the second gene was not retrieved as a significant hit. Possible regions of synteny were assayed in the genomic surroundings of the *GS2* isoforms. Syntenic regions, which are indicative of duplication events (Tang et al. 2008), were not observed in the genomic regions flanking the *GS2* isoforms (Supplementary Fig. S2). Therefore *GS2.1* is unlikely a result of a duplication event of *GS2*.

By producing a homology model of *A. palmeri* GS1, and docking GFA into its binding site, we identified seven amino acids involved in GFA binding: E131, E192, G245, H249, R291, R311 and R332 in GS1 (Supplementary Fig. S3). Their homologs in GS2 are E190, E251, G304, H308, R350, R370 and R391. Alignment of peptide sequences of *A. palmeri GS* isoforms with 34 other GSs (representing different plant families including Fabaceae, Malvaceae, Brassicaceae, Poaceae, and Asteraceae) showed full conservation at these positions, suggesting that mutations at the substrate-binding residues are not tolerable (brown rectangles in Fig. S1). Mutations at the substrate-binding residues of GS1 and GS2 rendered inactive or severely impaired protein in a *E. coli*-based assay (A. Porri, unpublished data), corroborating this hypothesis.

RACE primers were used for the amplification and sequencing of the untranslated regions (UTR). The lengths determined were: g13234: 5'UTR 83 bp, 3'UTR 268 bp; g1417: 5'UTR 77 bp, 3'UTR 67 bp; g17050: 5'UTR 132 bp, 3'UTR 163 bp. The *GS2.1* isoform was not detected in this experiment, indicating that it might be an unexpressed pseudogene (Chandrasekaran and Betrán 2008).

### Sequence analysis of *GS* isoforms in GFA survivors

Overall, all GS isoforms in 17 GFA survivors from MO#2 population showed a high level of conservation. Few mutations were detected in *GS1.1* and *GS1.2* (Supplementary Fig. S4 to S6). *GS2* from all 17 plants showed 100% sequence identity to the wild-type (WT), which illustrates the importance of this isoform in plant metabolism and the ‘fixed’ configuration of its catalytic site. The most prevalent mutation was N41D, found in *GS1.1* of six plants. In this same isoform, four mutations were detected once (G27D, Y95N, V109D and E122K) and N109Y was detected twice. In the *GS1.2* isoform, only three mutations were detected: D173E was found in three plants, and F114I and I220L were found only once.

### Copy number, transcript abundance and protein levels of GS isoforms in GFA-resistant plants

**Fig. 3:**
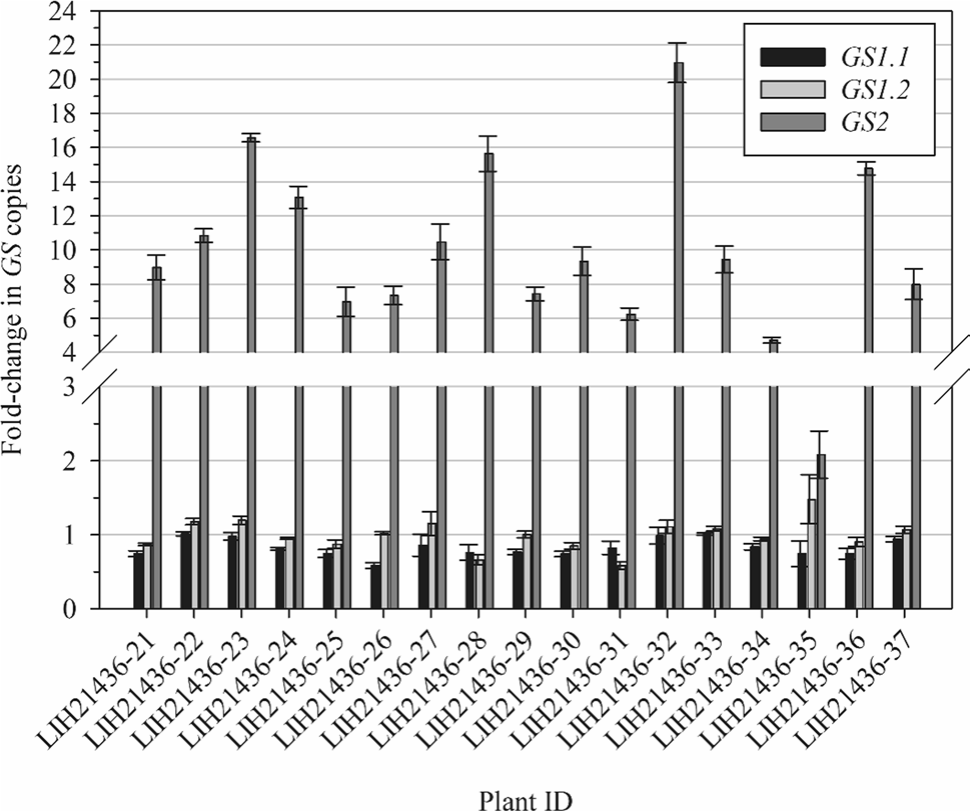
Fold-change in *GS* copies of 17 GFA survivors from the MO #2 population in relation to nontreated plants from a sensitive population. Fold-change was calculated using the 2^–∆∆Ct^ method as described in Schmittgen and Livak (2008), using Actin as internal control. Bars represent means and lines represent the standard error of the mean (*n* = 3 technical replicates)

**Fig. 4:**
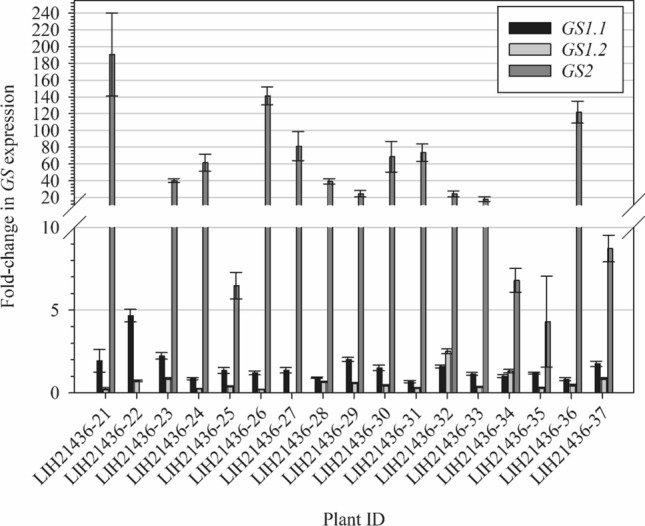
Fold-change in *GS* expression of 17 GFA survivors from the MO #2 population in relation to nontreated plants from a sensitive population. Fold-change was calculated using the 2.^–∆∆Ct^ method as described in Schmittgen and Livak (2008), using Actin as internal control. Bars represent means and lines represent the standard error of the mean (*n* = 3 technical replicates)

**Fig. 5:**
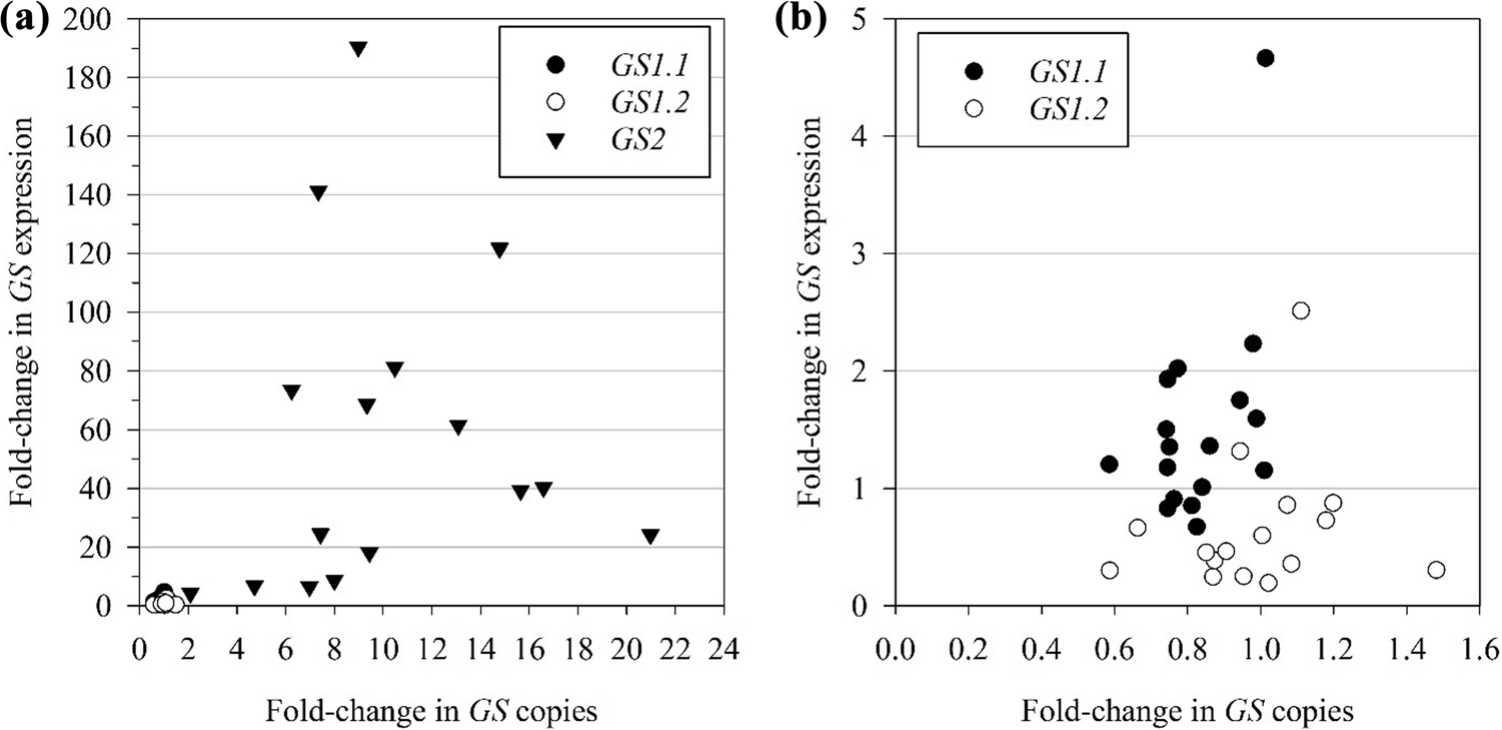
Correlation between fold-change in *GS* copies and expression, in 17 GFA survivors from the MO #2 population (**a**). Data points from *GS2* isoform were excluded from **b** for better visualization of data distribution

None of the samples showed increased copy of *GS1.1*, while only one sample showed increase in *GS1.2* copies (Fig. 3). On the other hand, 16 out of 17 samples showed a fourfold or higher increase in *GS2* copies. The highest copy number was observed in sample #32, where a 21-fold increase was detected. Fold change in *GS* expression followed a similar pattern: while both *GS1* isoforms had minimal or no increase in expression, *GS2* had a significant overexpression in all samples (Fig. 4). The lowest and highest fold change of *GS2* expression was 4- and 190-fold, respectively. There was no linear correlation between fold-change in expression and copy number of any of the isoforms studied. The isoform *GS1.1* was slightly over-expressed in some samples despite the absence of gene copy amplification (Fig. 5).

**Fig. 6:**
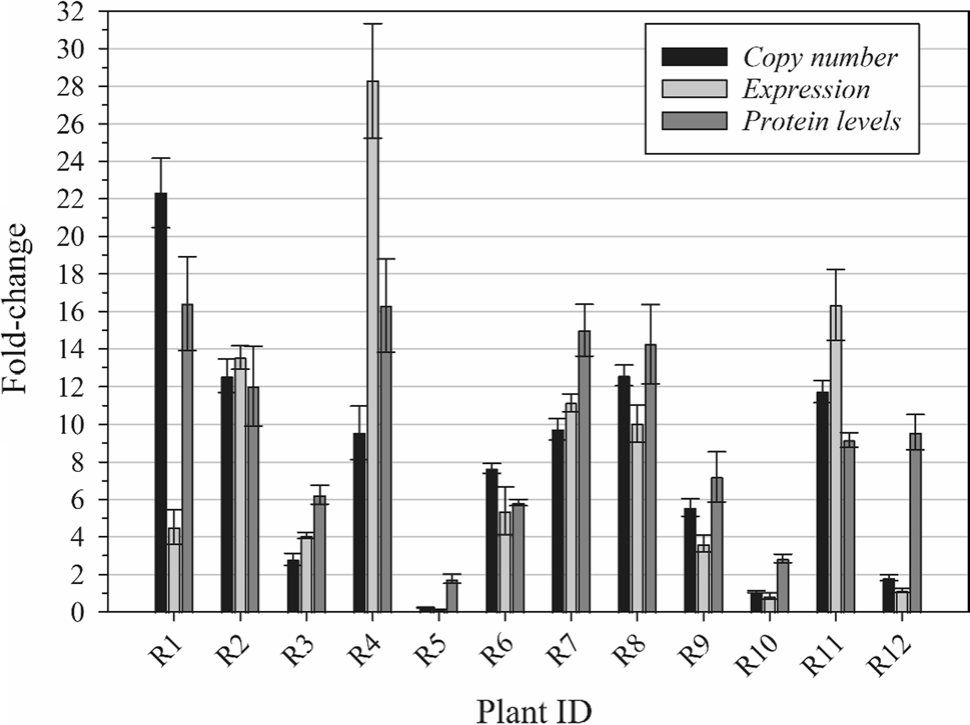
Fold-change in *GS2* copies, expression, and protein levels on 12 plants from the MO #2 F1 population compared to three plants from an SS population. Fold-change in *GS2* copies and expression was calculated using the 2^–∆∆Ct^ method as described in Schmittgen and Livak (2008), using Actin as internal control. Fold-change in GS2 protein levels was calculated by dividing the LFQ intensity of the sample by the average of three SS plants. Bars represent means of three technical replicates and lines represent the standard error of the mean

In 12 survivors from the MO#2 F1 population, fold-changes in copy number, transcript abundance and protein levels were determined relative to three plants from the SS population. Although GS1.1 and GS1.2 were detected at similar amounts in R and S plants (data not shown), GS2 levels were higher in all samples, with a minimum and maximum of 2- and 16-fold change, respectively (Fig. 6). As seen previously in the dataset produced from 17 plants from the field population, the correlation between gene copies, transcript abundance, and protein levels is weak, reinforcing the hypothesis that epigenetic or post-transcriptional mechanisms may play important roles in GS2 biosynthesis regulation in this resistant population.

### Ammonia quantification using a leaf-disc assay

**Fig. 7:**
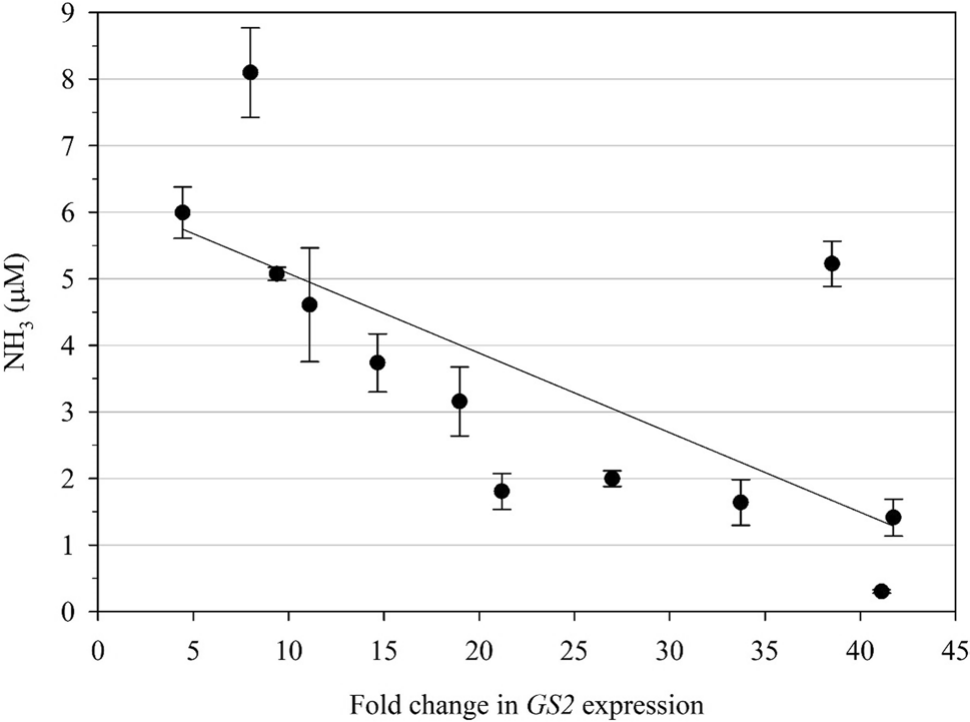
Correlation between ammonia accumulation and *GS2* expression fold-change relative to susceptible plants. Ammonia accumulation was determined spectrophotometrically at a wavelength of 630 nm. Absorbance was measured from leaf discs of 12 GFA-resistant plants incubated in 20 mM GFA solution. Bars represent the standard error of the mean (*n* = 3 technical replicates)

Ammonia accumulation is one of the physiological consequences of GS inhibition by GFA, and it has been used as a marker of plant susceptibility to this herbicide (Downs et al. 1994; Dayan et al. 2015). Therefore, plants with higher *GS* expression are expected to accumulate less ammonia. Twelve survivors from the MO#2 F1 population were submitted to an in vitro ammonia accumulation assay and had their *GS2* expression analyzed. As expected, there was a significant negative correlation between these two variables (r = -0.712, *P* = 0.00934), as seen in Fig. 7. One sample deviated from the prevailing pattern, showing higher ammonia levels compared to plants with similar fold-change in GS2 expression. This deviation is reflected in the lack of correlation in some samples between GS2 expression and protein content.

### Ectopic expression of *A. palmeri* GS2 in *N. benthamiana* leaf disc

**Fig. 8:**
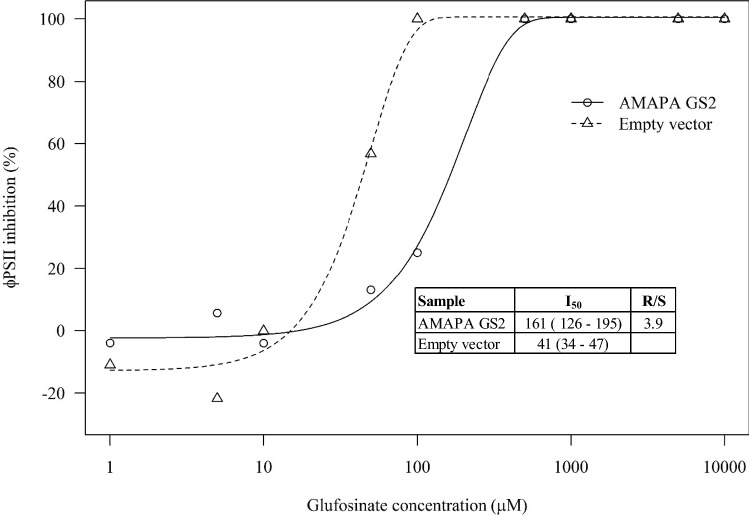
Response of *N. benthamiana* leaf-discs transiently overexpressing *A. palmeri GS2* or an empty vector to incubation in increasing concentrations of GFA. Photosystem II operating efficiency (ϕPSII) was obtained through chlorophyll fluorescence analysis and normalized to percent inhibition in relation to controls incubated in water. A Weibull II model was used to estimate I_50_ values. Confidence intervals of these parameters are shown between brackets

*Amaranthus palmeri GS2* was transiently overexpressed in *N. benthamiana* leaves, and an in vitro assay was performed by incubating leaf-discs in GFA solutions of increasing concentrations. The ϕPSII was determined and used as an indicator of the effect of GFA on photosynthetic activity. Samples that received the empty vector had an estimated I_50_ of 40 µM GFA, while samples that overexpressed the *A. palmeri GS2* showed a four-fold increase of that parameter (I_50_ = 160 µM) (Fig. 8). These results strongly suggest that overexpression of *GS2* is enough to increase plant tolerance to GFA.

## Discussion

In the present work, we describe the discovery of the first GFA-resistant *A. palmeri* genotype from Missouri, USA. In the greenhouse around 20% of plants from the MO #2 field population survived the labeled rate of GFA (657 g ha^−1^). The survival rate doubled in the progeny (MO #2 F1), equivalent to 6.1-fold resistance level relative to the susceptible population (Fig. 1). This level of resistance is already high, considering the frailty and uniformity of plant size under greenhouse conditions. The resistance problem is expected to be even higher in the field; hence, this field was noted as problematic, having a high number of escapes during the sampling year. GFA would be less effective, or could be inconsistent, under field conditions because of various mitigating factors including the large variability in plant growth stage and size; the ‘hardened’ condition of seedlings; sub-optimal environmental conditions around the time of herbicide application; and uneven spray coverage due to height differentials, plant crowding, and patchiness of plants. *Amaranthus palmeri* is a prolific seed producer and highly competitive (Ward et al. 2013). If these resistant plants are not controlled by other means, crop productivity will certainly be reduced (Massinga et al. 2001; Chandi et al. 2012) and the resistance problem to glufosinate will escalate. Hence, resistance to herbicides in general, and *A. palmeri* resistance to multiple herbicides (including GFA) in particular, is a threat to food security and economic sustainability.

Glutamine synthetase, the target of GFA, is a vital enzyme present in all living organisms. Enzymes in this family are classified as Type I, II or III based on its primary and quaternary structures (dos Santos Moreira et al. 2019). In plants, GS can be further categorized according to their subcellular location: GS1 enzymes are cytosolic, and GS2 enzymes are plastidic (Bernard and Habash 2009). While GS2 is encoded by a single, nuclear gene, GS1 is encoded by a multigene family generally composed of three to five isoforms (Swarbreck et al. 2010). In the present study, two cytosolic and one plastidic isoforms were discovered in the *A. palmeri* genome. The phylogenetic analysis of GS isoforms from 11 species (three monocots and eight dicots) showed a high similarity of *GS1.1*, *GS1.2* and *GS2* from *A. palmeri* with its respective homologs in *Spinacia oleracea* and *Chenopodium quinoa*, species that are also in the Amaranthaceae family (Fig. S1). *GS2* sequences from all species fall into a well-defined clade, as the divergence of cytosolic and plastidic *GS* genes pre-dates the divergence of monocots and dicots (Biesiadka and Legocki 1997). The three monocots grouped together regardless of the isoform considered. With the clear distinction of GS between dicots and monocots, the involvement of GS in resistance to glufosinate in these two groups of species may differ. This question will remain until other cases of resistance to glufosinate evolve and the resistance mechanism identified.

With the homology model of *A. palmeri* GS1.1 produced using *Z. mays* GS1 (PDB 2D3A) as a template, we identified seven residues involved in GFA binding: E131, E192, G245, H249, R291, R311, and R332. The same amino acids were found to interact with methionine sulfoximine (another glutamate analog) in *Z. mays* GS1 (Unno et al. 2006), and are homologous to E190, E251, G304, H308, R350, R370 and R391 in *A. palmeri* GS2. Interestingly, no polymorphisms were observed in these loci in any of the 34 sequences included in the phylogenetic analysis (brown rectangles in Fig. S1), highlighting their importance to proper protein function (Unno et al. 2006; Capra and Singh 2007). In accordance with that, mutations introduced in silico at the above-mentioned positions from *GS1.1* and *GS2* produced either inactive or severely impaired enzymes, based on an in vitro assay (A. Porri, unpublished data).

Allosteric interactions are known to be one of the regulatory mechanisms of GS activity (Stadtman 2001). Therefore, mutations outside the binding pocket could allosterically interfere with GFA binding and lead to herbicide resistance. To exclude that possibility, the three GS isoforms of 17 GFA survivors from the MO#2 population were sequenced. A total of six and three mutations were found in *GS1.1* and *GS1.2* isoforms, respectively, while no polymorphisms in *GS2* were detected in any of the 17 samples analyzed. The most prevalent mutation among GFA survivors was a N41D substitution in *GS1.1* (found in 6 out of 17 plants), but polymorphisms at that position are common (indicated by the pink rectangle in Fig. S1). The S59G substitution recently reported to confer GFA resistance in *E. indica* (Zhang et al. 2022) was not found in this experiment (blue rectangle in Supplementary Fig. S4 – S6). As no mutations were ubiquitous across the survivors analyzed, we conclude that GFA resistance in this *A. palmeri* genotype is not conferred by target-site mutations.

The *GS* copy number and expression level were also determined using the same 17 plants analyzed for *GS* polymorphisms. All isoforms (*GS 1.1*, *GS 1.2* and *GS2*) were assayed. *GS2* amplification was detected in all samples, but *GS1.1* and *GS1.2* copies were not augmented. Fold-change in transcript levels followed a similar pattern, with *GS1* isoforms showing little to no increase in expression and *GS2* being overexpressed to a great extent. It is intriguing that the correlation between *GS2* fold change in copy number and expression was weak (Fig. 5), suggesting that transcriptional regulation mechanisms might be involved in *GS2* overexpression. Similarly, the increase in GS2 protein levels were not always correlated with the fold-change increase in transcript abundance (Fig. 6), which points to a complex regulatory system of this biochemical pathway. In a few cases, such as with R5 and R10, resistance to GFA could not be attributed to GS2 gene amplification, overexpression, increased protein production, or target site mutation. Such resistant plants may harbor the ability to metabolize GFA faster than SS plants. This aspect is yet to be investigated. Meanwhile. changes in the methylation status of DNA and histones, as well as post-transcriptional mechanisms such as increase in mRNA stability are being investigated. It is possible that *GS2* expression is induced upon exposure to GFA. Corroborating this hypothesis, MO#2 plants that survived a 1 × GFA application were not killed by a second application of 4 × GFA applied 2 week later (S. Bowe, unpublished data).

The negative correlation between *GS2* expression and ammonia content in *A. palmeri* leaf-discs, and the increased GFA tolerance observed in *N. benthamiana* overexpressing GS2 present strong evidence that this naturally evolved mechanism confers resistance to GFA in *A. palmeri*. GS overproduction in transgenic rice (Cai et al. 2009; James et al. 2018), tobacco (Eckes et al. 1989), wheat (Huang et al. 2005) and poplar (Pascual et al. 2008), all resulted in GFA tolerance at the plant level. The same was observed at the cell level in tobacco (Ishida et al. 1989) and alfalfa (Donn et al. 1984). The irreversible nature of GS inhibition by GFA matches very well with resistance through target-site overproduction. The enzyme abundance not only allows the biochemical pathways to be maintained, but also reduces the pool of available herbicide molecules with time. Furthermore, gene amplifications can facilitate evolution by reducing the selective constraints in one or more copies (Flagel and Wendel 2009; Panchy et al. 2016). In other words, resistance-conferring mutations that would not be tolerated due to a strong fitness cost might be able to evolve as remaining copies are still functional. The close proximity of *GS2* and *GS2.1* provokes questions related to the evolution of this genomic region. We suggest that it is unlikely that these isoforms originated from a duplication event. Expression of *GS2.1* was not detected in plants in normal physiological conditions, but assessing the effect of abiotic stresses on *GS2.1* expression would be an interesting follow-up study.

Among the naturally evolved mechanisms of herbicide resistance, target-site amplification is rare (Gaines et al. 2020). The first and most notable example is the glyphosate-resistant *A. palmeri* carrying increased *EPSPS* copies (Gaines et al. 2010). Interestingly, glyphosate tolerance in carrot cell lines was attributed to an increase in EPSPS activity at least 25 years prior the discovery of this mechanism in *A. palmeri* (Nafziger et al. 1984). This adaptation mechanism can now be found in at least eight weed species as a result of convergent evolution (Patterson et al. 2017). Resistance by target site amplification can also be introgressed into other genomically compatible species via pollen flow such as what occurred between *A. palmeri* and *A. spinosus* (Nandula et al. 2014).

*EPSPS* copies are distributed throughout the genome of *A. palmeri* due to self-replication of the *EPSPS cassette*, a ~ 300 kbp circular extra-chromosomal DNA structure that carries 58 genes plus the *EPSPS* gene itself ( Gaines et al. 2010; Molin et al. 2017, 2020). Due to the large size and high copy number of the *EPSPS cassete*, genome size was shown to be up to 13% larger in R plants compared to S (Molin et al. 2017). Genome size analysis of plants from the MO#2 population did not detect any changes compared to plants from a SS population (M.M. Noguera, unpublished data). In *Bassia scoparia*, *EPSPS* copies are in tandem arrangement in a single chromosomal locus, likely originated from repeated unequal crossover (Jugulam et al. 2014). A greater understanding of the origin of these duplication events may facilitate the development of risk-prediction models, allowing proactive identification of ‘high risk’ species-by-chemistry combinations. Lastly, target-site amplification was also found in a *Digitaria sanguinalis* biotype cross-resistant to ACCase inhibitors (Laforest et al. 2017), but detailed information about its origin and distribution are not available.

In conclusion, our data strongly support the hypothesis that *GS* amplification and overexpression (particularly the plastidic isoform, *GS2*) is the main factor conferring resistance to GFA in this *A. palmeri* genotype. The co-occurrence of increased copy and increased expression of a herbicide target gene in the same plant is a novel adaptation mechanism that has not been detected previously. We hypothesize that epigenetic and post transcriptional mechanisms are likely to contribute to the overproduction of GS2 at the protein level, as these mechanisms are known to promote quick changes in transcript synthesis and translation (Floris et al. 2009; Van Ruyskensvelde et al. 2018; Zhang et al. 2018). Follow-up studies include determination of the distribution of *GS2* copies throughout the genome, the elucidation of the mechanism of *GS2* amplification, possible transcriptional and post-transcriptional regulation mechanisms involved in overexpression and protein synthesis, and contribution of additional traits towards resistance (such as ability to metabolize GFA, reduced absorption/translocation, or increased protection against oxidative damage). The multiple layers of regulation of protein biosynthesis in plants poses a challenge in elucidating herbicide resistance mechanisms related to target site overproduction, and the MO#2 population is a clear example of that. The history of *A. palmeri* adaptation to herbicide selection pressure shows that its management must not rely solely on the chemical approach. The use of a diversified strategy should be practiced, such as crop rotation, tillage, the use of preemergence herbicides, precise application time at young plant stage and herbicide mixtures of complementary mechanisms of action. The spread of GFA-resistant genotypes should be treated as a serious concern from the economical and humanitarian standpoints.

### *Author contribution statement*

Conceptualization of research project: M.M.N., N.R-B., A.P., J.H., and J.L. Bioinformatic analysis: J.L., B.M., and M.M.M. Sequencing of GFA survivors: F.B. Homology modelling: M.B. Greenhouse-related activities: M.M.N, I.S.W., and L.M. Protein quantification: F.G., M.P., and C.T. Data analysis: M.M.N., A.P., J.L., B.M., M.B., F.G., M.P., and C.T. Manuscript preparation and review: M.M.N., A. P., N.R-B, I.S.W., J.H., J.L., B.M., M.B., F.G., M.P., and C.T.

## Supplementary Information

Below is the link to the electronic supplementary material.

Supplementary file1 (DOCX 1884 KB)

## Data Availability

The datasets generated during and/or analyzed during the current study are available from the corresponding author upon request.

## Abbreviations

GFA: Glufosinate-ammonium
GS: Glutamine synthetase
SS: Sensitive standard
