# Supplementary material for: Involvement of glutamine synthetase 2 (GS2) amplification and overexpression in Amaranthus palmeri resistance to glufosinate
Source: Planta. 2022 Aug 12;256(3):57. doi: 10.1007/s00425-022-03968-2 (PMC9374794; doi:10.1007/s00425-022-03968-2)
Supplement: Supplementary file 1 — Supplementary file1 (DOCX 1884 KB) [file 425_2022_3968_MOESM1_ESM.docx]

**Supplementary file F1.** Genbank/Phytozyme entries used for construction of phylogenetic tree.

*A.thaliana*_GS1;1 (NP_198576.1);

*A.thaliana*_GS1;2 (NP_176794.1);

*A.thaliana*_GS1;3 (NP_188409.1);

*A.thaliana*_GS1;4 (AED92312.1);

*A.thaliana*_GS1;5 (NP_175280.1);

*A.thaliana*_GS2 (NP_001031969.1);

*C.quinoa*_GS1.1 (XP_021757390.1);

*C.quinoa*_GS1.2 (XP_021724688.1);

*C.quinoa*_GS2 (XP_021727743.1);

*G.hirsutum*_GS1.1 (XP_016737511.1);

*G.hirsutum*_GS1.2 (XP_016696747.1);

*G.hirsutum*_GS2 (XP_016670596.1);

*L.sativa*_GS1.1 (XP_023770002.1);

*L.sativa*_GS1.2 (XP_023754618.1);

*L.sativa*_GS2 (XP_023733962.1);

*N.tabacum*_GS1 (XP_016466322.1);

*N.tabacum*_GS2 (XP_016440217.1);

*O.sativa*_GS1-1 (XP_015626102.1);

*O.sativa*_GS1-2 (XP_015631679.1);

*O.sativa*_GS1-3 (XP_015628694.1);

*O.sativa*_GS2 (XP_015635322.1);

*P.vulgaris*_GS1b (XP_007152660.1);

*P.vulgaris*_GS1g (XP_007141923.1);

*P.vulgaris*_GS2 (XP_007147796.1);

*S.oleracea*_GS1.1 (Spov3_chr5.00751);

*S.oleracea*_GS1.2 (Spov3_chr4.02946);

*S.oleracea*_GS2 (Spov3_chr3.03310);

*S.viridis*_GS1.1 (Sevir.1G317300.1.p);

*S.viridis*_GS1.2 (Sevir.9G117000.1.p);

*S.viridis*_GS1.3 (Sevir.9G489700.1.p);

*S.viridis*_GS2 (Sevir.3G024800.1.p);

*Z.mays*_GS1.1 (BAA03432.1);

*Z.mays*_GS1.4 (AFP20991.1);

*Z.mays*_GS2 (NP_001352144.1).

**Table S1.** Primers used for sequencing and copy number/expression analysis of *GS* isoforms from *A. palmeri*. Fluorescent dyes for qPCR probes are shown in the footnote.

| **Objective** | **Name** | **Sequence (5' > 3')** | **Ta (°C)** |
| --- | --- | --- | --- |
| Sequencing | GS1.1-F | GAAGAACATACTCATCTTCCACTTCTC | 63 |
|  | GS1.1-R | TGCACAATAATGGCAGAGAAGATC | 63 |
|  | GS1.2-F | TCTTCGTATTCTCTTTCATCTATGTCC | 53 |
|  | GS1.2-R | CCAAGAAATTCCAAATTCACATTAACA | 53 |
|  | GS2-F | CGACCACCCTTTTCCGATCA | 60 |
|  | GS2-R | TGGGCACGTGAAASAGTTCC | 60 |
| qPCR | GS1.1-F | TGTGTGATGCCTATACTCCACA | 60 |
|  | GS1.1-R | TACCATGGTTCCTCGGCAAC | 60 |
|  | GS1.1-probe^1^ | AGGAGAGCCAATCCCAACCAACA | 60 |
|  | GS1.2-F | TGTGTGATGCATACACCCCG | 60 |
|  | GS1.2-R | GACGTCGGGATGGCTAAAGA | 60 |
|  | GS1.2-probe^2^ | GCTGGAGAACCAATTCCAACAAACAAG | 60 |
|  | GS2-F | TGGCACAAATACTTGCACCTT | 60 |
|  | GS2-R | GCTGCTCCACCCTGTTTACT | 60 |
|  | GS2-probe^1^ | AGGCTCCACAAGTTCAATGACATCAA | 60 |
|  | Actin-F | GCGGAAAGCTAAGCGTGAAC | 60 |
|  | Actin-R | TCAGACCTGCTCTGGAGTCA | 60 |
|  | Actin-probe^3^ | GGAGGAAAAGGCGGATGCTGCA | 60 |

^1^Fam/BMN-Q535

^2^Cy5/BMN-Q650

^3^Hex/BMN-Q535


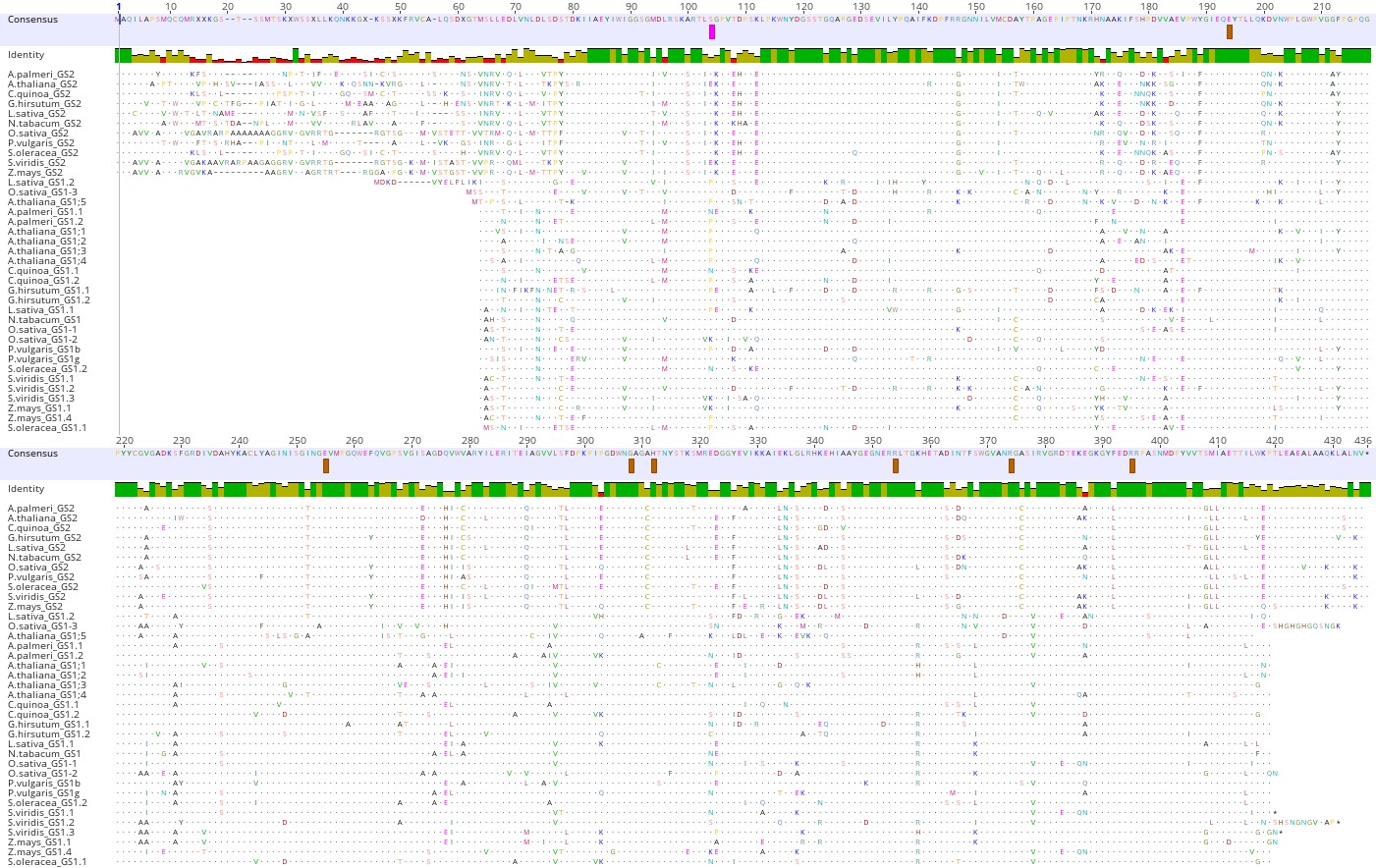
**Fig S1.** Multiple alignment of *A. palmeri* *GS* isoforms with 34 other *GS* sequences representing 11 plant species. Pink rectangle under the consensus sequence indicates the 41^st^ position *of A. palmeri GS1.1*, where polymorphisms were detected in 6 of 17 survivors from MO #2 population. Brown rectangles indicate the amino acids involved in substrate-binding, which are highly conserved across species.


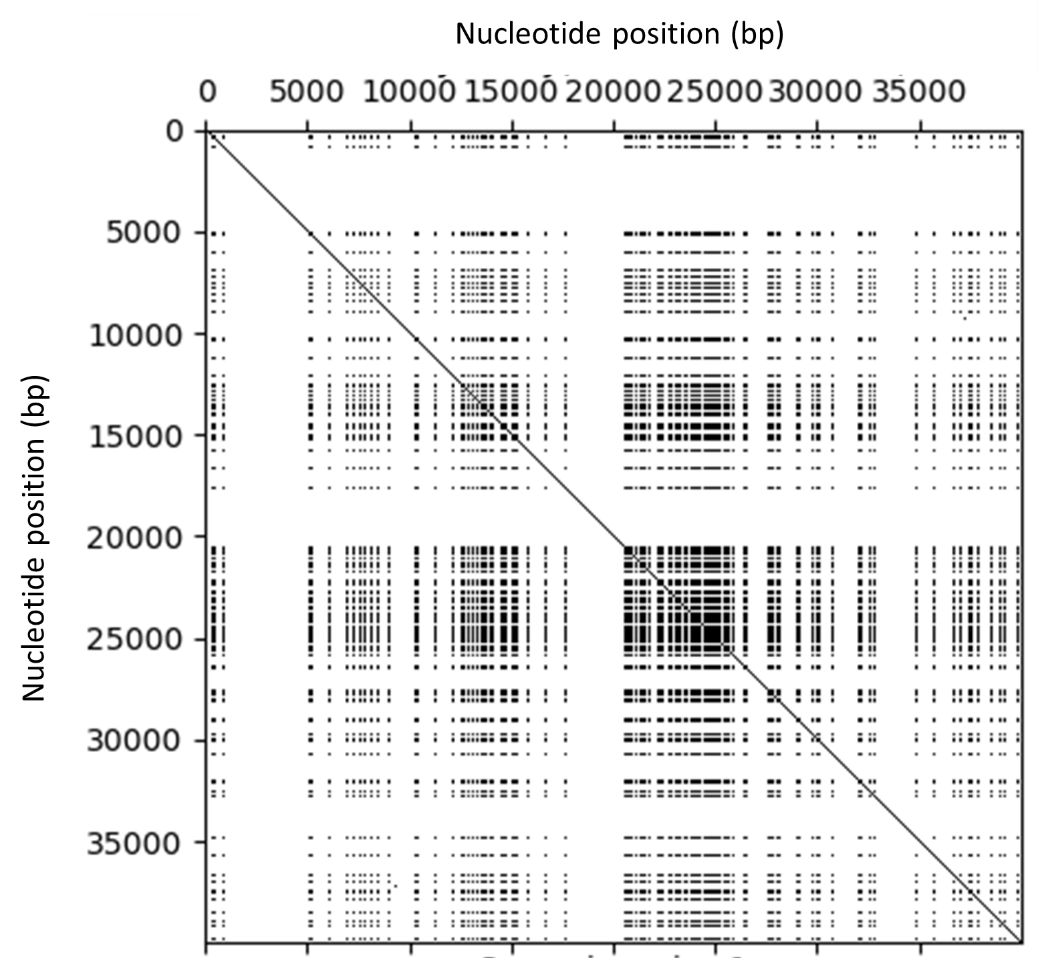


**Fig S2.** Nucleotide-to-nucleotide synteny plot of the genomic region containing *GS2.1* and *GS2*. Diagonal lines are indicative of synteny, while vertical/horizontal lines are indicative of repeats. A window size of 40 kb and kmer size of 10 bp was used.

**
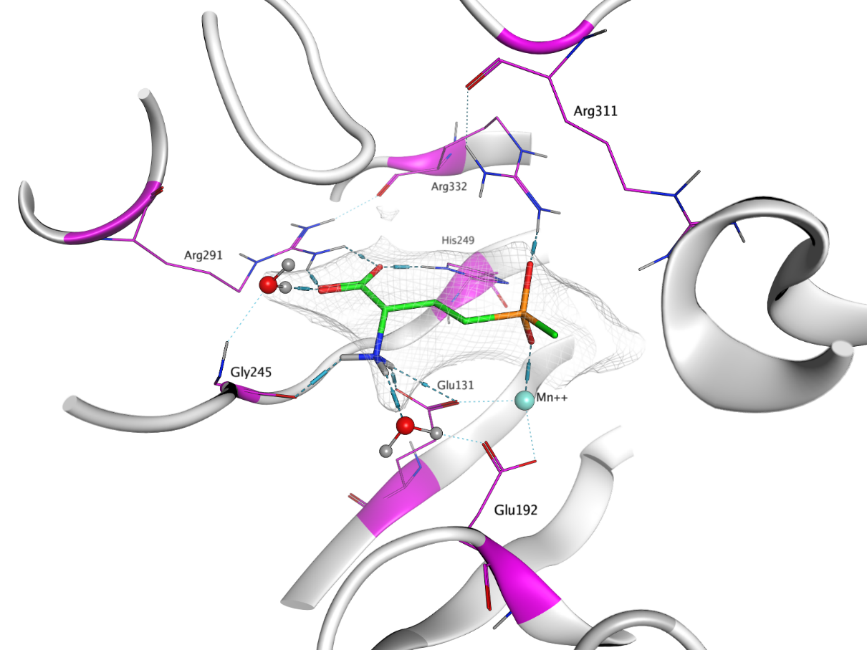
Fig S3.** Homology model of *A. palmeri* GS1 with a GFA molecule bound into its catalytic site. The seven amino acids directly involved in GFA-binding are highlighted in fuchsia. Sticks in GFA molecule are color-coded: green = C, orange = P, red = O, blue = N and grey = H.


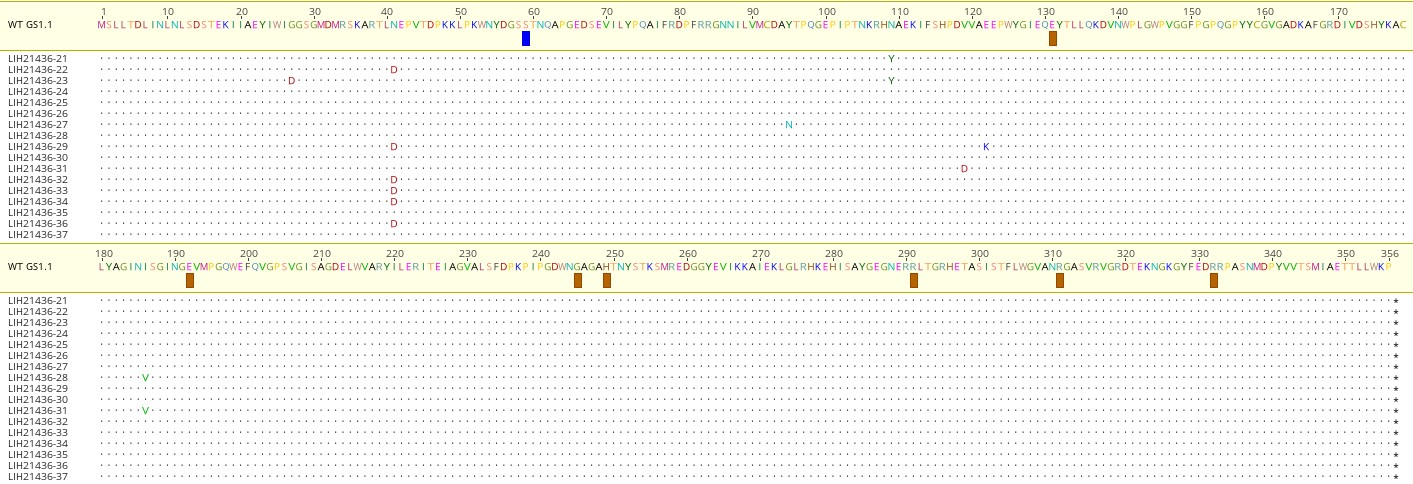
**Fig S4.** Multiple alignment of *GS1.1* sequences of 17 GFA survivors. Brown rectangles indicate substrate-binding residues, and the blue rectangle locates S59, where a mutation was reported to confer resistance to GFA in *Eleusine indica* ^1^.


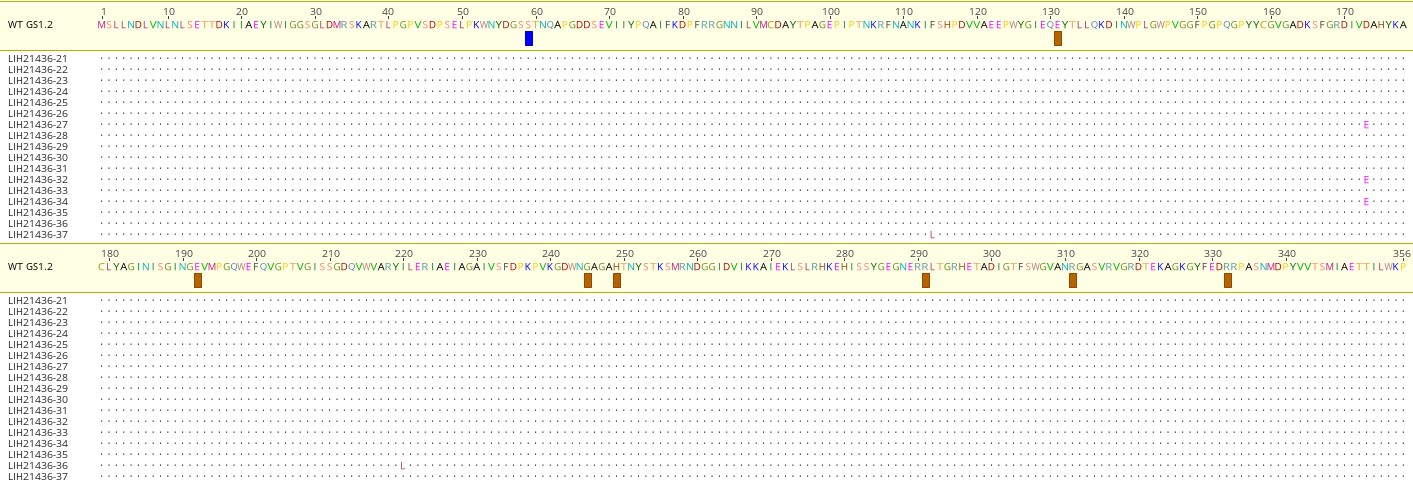


**Fig S5.** Multiple alignment of *GS1.2* sequences of 17 GFA survivors. Brown rectangles indicate substrate-binding residues, and the blue rectangle locates S59, where a mutation was reported to confer resistance to GFA in *Eleusine indica* ^1^.


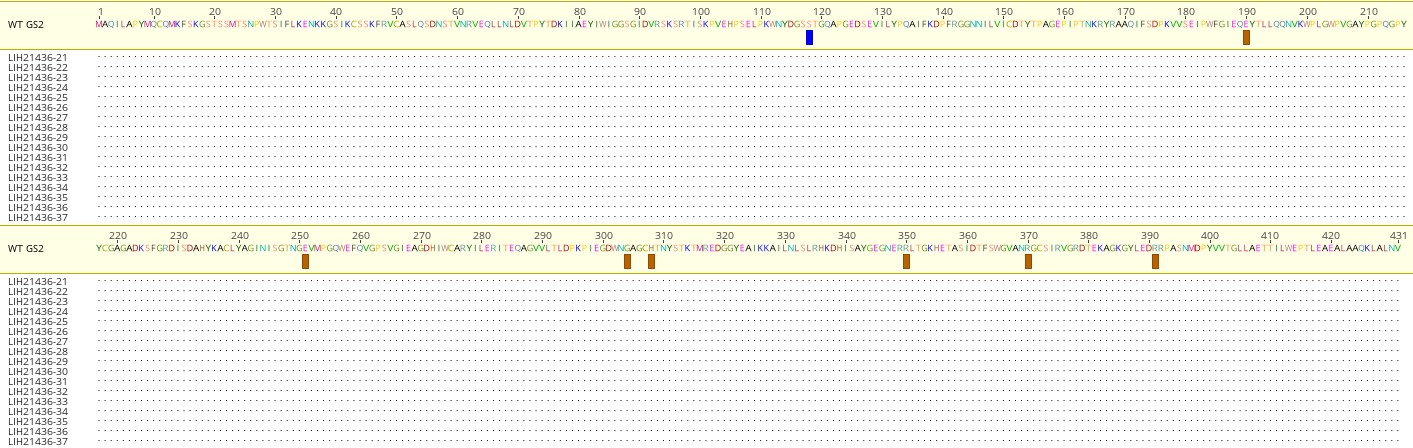


**Fig S6.** Multiple alignment of *GS2* sequences of 17 GFA survivors. Brown rectangles indicate substrate-binding residues, and the blue rectangle locates the residue homologous to S59, where a mutation was reported to confer resistance to GFA in *Eleusine indica* ^1^.

1. Zhang C, Yu Q, Han H, Yu C, Nyporko A, Tian X, et al., A naturally evolved mutation (Ser59Gly) in glutamine synthetase confers glufosinate resistance in plants. *Journal of Experimental Botany* 2022).
